# RTN1 and RTN3 protein are differentially associated with senile plaques in Alzheimer’s brains

**DOI:** 10.1038/s41598-017-05504-9

**Published:** 2017-07-21

**Authors:** Qi Shi, Yingying Ge, Wanxia He, Xiangyou Hu, Riqiang Yan

**Affiliations:** Department of Neurosciences, Lerner Research Institute, Cleveland Clinic, Cleveland, OH 44195 USA

## Abstract

Reticulon proteins (RTNs), consisting of RTN1 to RTN4, were previously shown to interact with BACE1 by negatively modulating its secretase activity. In RTN3-null mice, RTN1 expression was slightly elevated. To understand the *in vivo* role of RTN1, we generated RTN1-null mice and compared the effects of RTN1 and RTN3 on BACE1 modulation. We show that RTN1 is mostly expressed by neurons and not by glial cells under normal conditions, similar to the expression of RTN3. However, RTN1 is more localized in dendrites and is an excellent marker for dendrites of Purkinje cells, while RTN3 expression is less evident in dendrites. This differential localization also correlates with their associations with amyloid plaques in Alzheimer’s brains: RTN3, but not RTN1, is abundantly enriched in dystrophic neurites. RTN3 deficiency causes elevation of BACE1 protein levels, while RTN1 deficiency shows no obvious effects on BACE1 activity due to compensation by RTN3, as RTN1 deficiency causes elevation of RTN3 expression. Hence, expression of RTN1 and RTN3 is tightly regulated in mouse brains. Together, our data show that RTN1 and RTN3 have differential effects on the formation of senile plaques in Alzheimer’s brains and that RTN3 has a more prominent role in Alzheimer’s pathogenesis.

## Introduction

The reticulons (RTNs) are a protein family with a characteristic C-terminal membrane-bound reticulon-homology domain (RHD)^1–3^. In mouse and human, the RTN family consists of four members, RTN1 to RTN4; each of which has multiple spliced variants due to the use of alternative exons or translation initiation codons. The N-terminal sequences among RTN members are completely divergent, while the C-terminal RHD is highly conserved. The RHD domain in mouse and human is organized such that there are two transmembrane-anchoring stretches separated by a 66 amino acid-long loop and a short C-terminal tail. This domain is required for localizing RTN on the endoplasmic reticulum (ER) membrane and for shaping tubular ER structure^4, 5^.

Our interest in RTNs dates back to our discovery of their roles in negative modulation of Alzheimer’s β–secretase^6^, which has also been validated by others^7, 8^. This β–secretase, known as BACE1 for β-site amyloid precursor protein (APP) cleaving enzyme 1^9^, is indispensable for the generation of β-amyloid peptide (Aβ)^10^. BACE1 initiates cleavage of APP to release a membrane-bound, 99 amino acid-long C-terminal fragment (APP-C99), which is further cleaved by γ-secretase to excise Aβ^11^. We and others have shown that increased expression of any RTN member in cells leads to reduced BACE1 activity and Aβ generation^6–8^. Our previous study focused on RTN3, which is richly expressed by neurons, although RTN3 is also ubiquitously expressed in all other cells or tissues to a lesser extent^12^. Mice overexpressing RTN3 in neurons show reduced Aβ generation and amyloid deposition^13, 14^. However, the effects of RTN3 deficiency on Aβ generation and amyloid deposition in mice is weak^12^, likely due to compensation by other RTN members in neurons. We indeed found slightly increased RTN1 expression in RTN3-null mice.

To better understand the role of RTN1 *in vitro* and *in vivo*, we generated RTN1-null mice using a conventional homology recombination approach, mainly because of the finding that RTN1 is largely expressed in brain^15^. Like RTN3-null mice, RTN1-null mice are also viable and show no obviously discernible abnormalities in growth or behavior. This RTN1-null mouse model has allowed us to characterize the expression of RTN1 in mouse tissues, and we demonstrate that RTN1 expression under normal conditions is restricted mainly to the nervous system and is significantly weaker in peripheral tissues such as spleen and lung. Although RTN1 is richly expressed in neurons similar to RTN3, it exhibits much weaker effects on BACE1 expression and is more weakly associated with dystrophic neurites in Alzheimer’s amyloid plaques. Our data suggest that RTN3 has a stronger association with Alzheimer’s disease (AD) pathology than RTN1, and this difference is likely related to unique sequences that are present in RTN1 and RTN3 as well as their differential localizations in neruites.

## Materials and Methods

### Creation of RTN1-null mice

Genomic DNA spanning the RTN1 gene was purchased from Invitrogen. The 5′ (5.4 kb) and 3′ (2.8 kb) arms of DNA fragments, which flank exons 4 to 8 of the mouse RTN1 gene, were obtained by restriction enzyme digestion, then purified and sequentially subcloned into the targeting Neo/TK vector (as outlined in Fig. 2B). The cloned DNA fragments were confirmed by both restriction digestions and DNA sequencing. The generated targeting vector was then transfected into 129 SvEvTac ES cells by electroporation. Selection of positive ES cells was confirmed by Southern blot analysis and the targeting vector-integrated ES cells were injected into blastocysts derived from C57BL/6J ES cells. The injected blastocysts were then transferred into pseudo-pregnant female mice to give birth to chimeric mice, which were further bred with wild-type C57Bl/6 to generate RTN1 heterozygous and homozygous knockout mice. The founder mice were backcrossed with C57BL/6 mice and maintained in C57BL/6 congenic genetic background. Genotyping of mice was conducted by either PCR or Southern blotting, or both if necessary. The PCR primers for genotyping were as follows:

RTN1KO557-F, 5′- gaaaaggtgtgggcttttga -3′;

RTN1KO557-R, 5′- gcttggctggaggtaaactc -3′;

mRTN1WT660F, 5′- ccacgacggtgtttatgtga -3′;

mRTN1WT336R, 5′- accctgctgcttatgggtaa -3′.

All mice in the study were maintained and used according to the protocols approved by the Institutional Animal Care and Use Committee at the Cleveland Clinic Foundation.

### Cell lines and reagents

The APP overexpressing cell line (125.3 cell line) was generated by overexpressing Swedish mutant APP in HEK-293 cells as described in previous studies^6^. Antibodies against β-amyloid peptide (6E10; Catalog #: NE1003-100UL, RRID:AB_564201), calnexin (C4731, RRID: AB_476845), β-actin (A5441, RRID: AB_476744), and APP C-terminus (A8717, RRID: AB_258409) were purchased from Sigma-Aldrich (St Louis, MO). RTN1 and RTN3 antibodies were generated in the Yan lab. Antibodies R454 and R458 recognize the N-terminus and C-terminus of RTN3, respectively; R418 is an antibody specific to RTN1 C-terminus and recognizes all RTN1 isoforms. Alexa Fluor 488- and 568-labeled secondary antibodies were purchased from Invitrogen (Carlsbad, CA). DAB (3,3′ Diaminobenzidine Tetrahydrochloride, D5905) was purchased from Sigma-Aldrich. Avidin/Biotin Complex Elite Kits were purchased from Vector Laboratories (Burlingame, CA). Complete protease inhibitor tablets were purchased from Roche Biosciences (Palo Alto, CA). Bis–Tris NuPage gels (4–12%) were purchased from Invitrogen (Carlsbad, CA).

### Immunohistochemistry and immunofluorescent confocal microscopy

Immunohistochemical and confocal experiments were performed according to standard methods as previously described^12^. Frozen brain tissues from AD patients and mice were cut on a freezing microtome (Microm GmbH, Walldorf, Germany). For postmortem human brains, fixed sections were treated with 3% Sudan Black B for 10 min to remove autofluorescence. For mouse samples, hemi-brains of mice were routinely fixed with 4% paraformaldehyde and O.C.T. embedding and were sagittally sectioned at a thickness of 14 or 16 μm, while the other half of the brain was used for preparing protein lysates. Amyloid deposition was detected with monoclonal antibody 6E10 (Sigma, 1:1000); RTN1 immunoreactivity was detected with polyclonal R418 (1:1000); RTN3 immunoreactivity was detected with polyclonal R458 (1:1000); astrocytes were labeled by GFAP expression with monoclonal antibody Smi22 (1:1000, Covance). For immunohistochemical staining, we used the complex of HRP-conjugated secondary antibody and DAB staining. For confocal experiments, either Alexa Fluor 488- or Alexa Fluor 568-labeled secondary anti-IgG antibody (1:2000 dilution) was used for detecting fluorescent signals. Images were examined and captured with a Leica microscope.

### Western blot analysis of brain proteins

For western blotting, snap-frozen brain sections were homogenized on ice in 1% CHAPS extraction buffer containing complete protease inhibitors (Roche Bioscience) and 0.1 mM Na_3_VO_4_ for inhibiting phosphatase. The homogenates were rotated for 30 min at 4 °C to ensure extraction of membrane proteins. After centrifugation at 15,000 × g for 120 min, supernatants were collected and protein concentration was measured with the BCA protein assay reagent (Pierce). Equal amounts of lysate proteins were resolved on 4–12% bis–tris NuPage gels, followed by standard Western blotting with the antibodies specified above. Chemiluminescent signals were scanned and integrated density values were calculated with a chemiluminescent imaging system (Alpha Innotech, San Leandro, CA).

## Results

### Generation of mice deficient in RTN1 and analyses of RTN1-null phenotypes

**Figure 1:**
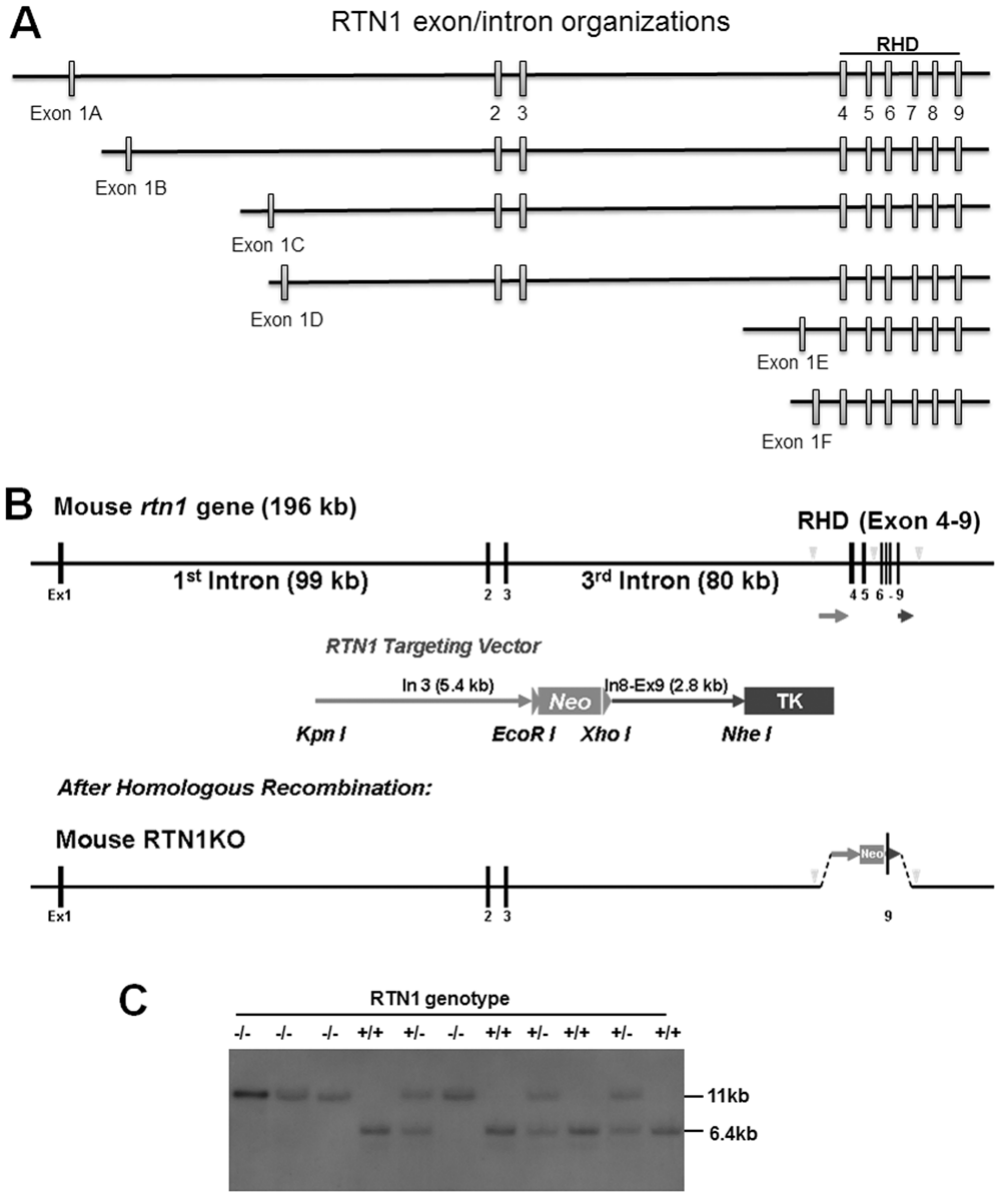
Generation of RTN1-null mice. (**A**) Schematic illustration of genomic structures of the mouse *rtn1* gene, with each box representing an exon. The exon size is not proportionally represented. Exon 1A to 1F contains the translation initiation codon. The reticulon homology domain (RHD) is encoded by exons 4 to 9. (**B**) Targeting DNA vector (the standard Neo/TK cassette) is generated by insertion of two arms: one 5.4 kb fragment from intron 3, and the other 2.8 kd fragment having a sequence spanning intron 8 to exon 9. The restriction enzymes used for generation these two fragments are illustrated. The arrowhead indicates the Eco RI site. (**C**) Southern blotting was performed to validate the founder lines of RTN1 knockout (KO) mice and subsequently generated RTN1-null mice. KO mice produce an 11 kb DNA fragment, while wild-type (WT) mice produce a 6.4Kb DNA fragment.

The initial reports of RTN1 indicated the presence of three RTN1 splicing variants^16, 17^. Further *in silico* annotations of EST databases revealed the presence of six different splicing variants with the potential use of six different translation start codons, with the isoforms share the common RHD encoded by exons 4 to 9 (outlined in Fig. 1A). Based on prior publications that suggest the use of A, B, C annotation for transcripts utilizing different translational initiation sites in a particular RTN member^18^, RTN1 isoforms are named from RTN1-A to RTN1-F; RTN1-A to RTN1-D contain exons 2–9, while RTN1-E and RTN1–F mainly contain exons 4-9.

To explore the physiological functions of RTN1, we generated RTN1-null mice. The mouse *rtn1* gene is 196 kb long and the longest form of RTN1-A contains all 9 exons; six exons coding for the highly conserved RHD domain are located in a short span compared to other coding exons and non-coding regions (Fig. 1B). Intron 1 contains three different initiation sites for producing RTN1 splicing variants RTN-1B, RTN-1C and RTN-1D, while intron 3 contains two additional initiation sites for producing RTN-1E and RTN-1F. For complete disruption of RTN1 function, we designed a targeting construct that removed exons 4–7 through homologous recombination and RTN1-null mice are expected to have the functional RHD domain deleted. For efficient recombination, a 5.4 kb fragment amplified from intron 3 and a 2.8 kb fragment spanning exons 8 and 9 were inserted between the *Neo* and *TK* (thymidine kinase) cassettes, respectively. The validated targeting vector was microinjected into 129 SvEvTac blastocysts for generating knockout mice. We confirmed properly targeted deletion of the *rtn1* gene in founder mice by both PCR genotyping and Southern blot analyses (Fig. 1C). Validated RTN1-null mice were maintained in the congenic C57BL/6 background by routine breeding strategies.

Like previous reports for RTN3-null^12^ and RTN4-null mice^19–21^, normal Mendelian inheritance was observed in the deletion of either one or both alleles of the *rtn1* gene. No obviously discernible abnormalities were detected in RTN1-null (RTN1^−/−^) mice at any age. Overall body weight during various growth stages as well as weights of various tissues such as the brain, heart, liver, pancreas, kidney, thymus, testis, and ovary showed no overt differences among the various genotypes. Although RTN proteins are thought to shape tubular ER, we found no significant alterations in ER tubular structure in our examinations of brain cells (data not shown), which is consistent with prior observations that deletion of one RTN member has no effect on overall tubular ER shape^5^. Altogether, we conclude that RTN1-null mice show no obvious growth defects.

### RTN1 is predominantly expressed in the mouse brain

**Figure 2:**
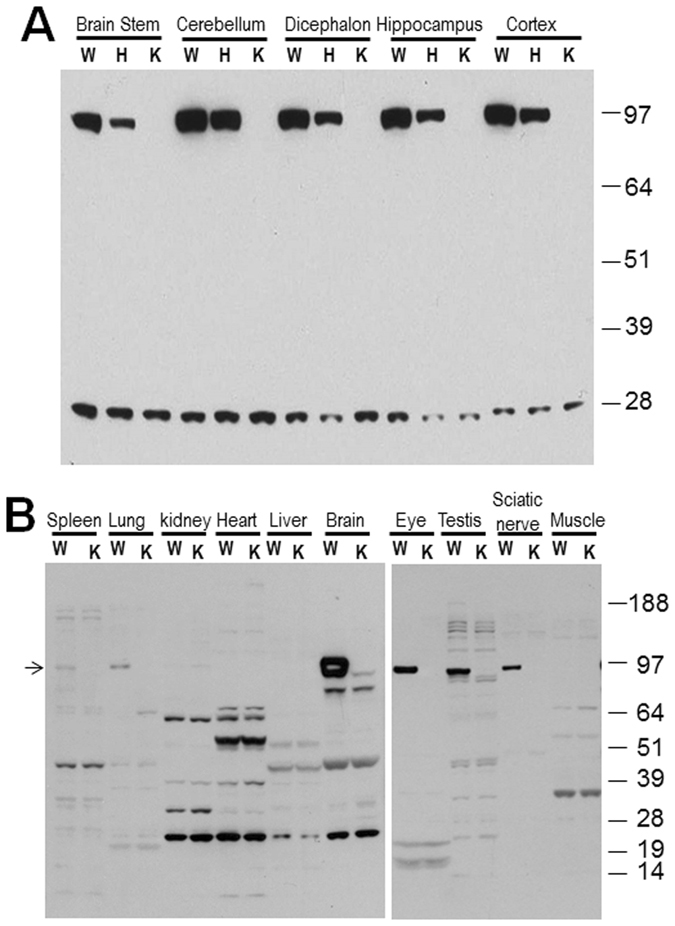
RTN1 deficiency increases RTN3 protein levels. (**A**) Protein lysates were prepared from different brain regions as indicated, and RTN1 was shown to be broadly expressed in all examined brain regions. This result shows mice at 6 months of age. Only RTN1-A was detected by antibody R418. A cross-reacted protein migrated near 25 kd and was detected in all WT, heterozygous, and homozygous RTN1-null mice. (**B**) Equal amounts of protein lysate from the indicated tissues were examined by the same procedure, and lysates from the brain cortex were used to locate the RTN1 isoform and to compare expression of RTN1 across different tissues. Arrow specifies the longest RTN1 isoform detected in brain. Non-specifically reactive bands would be sparse if more stringent western blot conditions such as those in panel A were used.

RTN1 was previously recognized as a neuroendocrine-specific protein^22^. The available RTN1-null mice allowed us to determine its expression and isoforms using the antibody R418, which recognizes the C-terminus of RTN1. In adult mice (6 months of age in this example), the band migrating at ~110-kd was broadly expressed in multiple brain regions and matches the mouse RTN1-A isoform, which encodes 776 amino acids (Fig. 2A). Surprisingly, no other isoform was detected, except for one band near 25 Kd as a non-specifically reacted band in all genotypes of mice. Expression of RTN1 in normal mice was significantly stronger in the brain when compared to other tissues (Fig. 2B), which is similar to the expression pattern of RTN3, which is richly expressed in brains and more weakly expressed in peripheral tissues^12^. Expression of RTN1 was also detected in sciatic nerves, testis, and eye, and was sparsely detected in lung and spleen. Other RTN1 splice variants were not detectable in this age group (Fig. 2B); cross-reacted bands present in both wild-type (WT) and RTN1-null mice suggested non-specifically reacted products. Although annotated and previously identified RTN variants were not readily detected in adult mice, we cannot exclude the possibility that these isoforms are expressed at significantly weaker levels or can be expressed in association with development, abnormal cellular growth, or diseased states. For example, increased expression of RTN1 was observed in the diseased mouse and human kidneys, and its expression correlates inversely with renal function in patients with diabetic nephropathy^23^. Hence, the available RTN1-null mice will be highly useful for future studies in various disease models.

### RTN1 is mostly expressed by neurons and is enriched in dendrites

**Figure 3:**
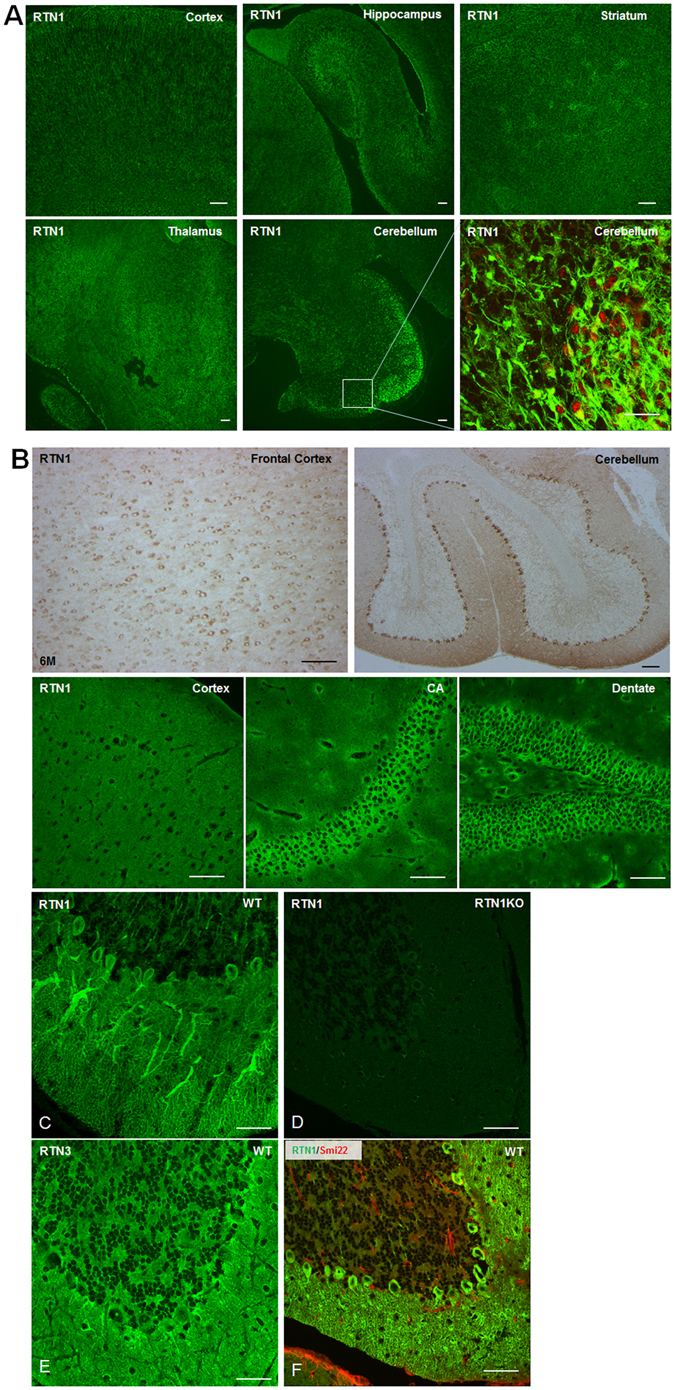
RTN1 is a neuronal protein. (**A**,**B**) Confocal and immunohistochemical staining of RTN1 in brain sections from postnatal day 0 (**A**) and at six months of age (**B**) were performed with antibody R418. RTN1 is clearly expressed in neurons in broad brain regions including the frontal cortex, cerebellum, and hippocampal CA and dentate gyrus. (**C**–**E**) Confocal results showed strong expression of RTN1 in Purkinje cells and dendrites by R418 staining (**C**), which was not seen in RTN1-null brain sections (**D**). RTN3 labels neurons, including Purkinje cells, but dendrites of Purkinje cells were not highlighted by RTN3 antibody (**E**). (**F**) Co-staining of RTN1 antibody (green) and Smil22 (red) were conducted to label astrocytes. Scale bar is 50 µm.

To determine the cellular expression of RTN1 in brain, we conducted immunohistochemical and confocal staining. We showed that RTN1 is strongly expressed in the neuronal cell body of all brain regions including cerebral cortex, hippocampus, and cerebellum in early development (examples of postnatal day 0, Fig. 3A) and in adult (examples of 6 month of age, Fig. 3B). Strikingly, in cerebellum, RTN1 had a strong localization in dendrites of mature Purkinje cells (Fig. 3C), and this staining is specific as antibody R418 showed no similar staining patterns in neurons or neuronal processes in RTN1-null brains (Fig. 3D). RTN3 was also clearly expressed, although relatively weakly, in Purkinje cells, but RTN3 had no evident presence in dendrites (Fig. 3E). Within the cerebellum, RTN1 is weakly expressed while RTN3 is strongly expressed by granule cells in the granular layer. This clear contrast in expression and localization patterns between RTN1 and RTN3 suggests that these two proteins have unique functional roles in neurons, despite the fact that both RTN1 and RTN3 have a highly conserved RHD, which presumably mediates the curvature of tubular ER.

Noticeably, RTN1 was not expressed by astrocytes, as no co-staining of RTN1 antibody with GFAP (Smi22) antibody was evident (Fig. 3F). RTN1 was also not found in microglia. Altogether, we showed that both RTN1 and RTN3 are highly expressed by neurons but display strikingly different neuritic localization patterns, with RTN1 being significantly more enriched in dendrites.

### The impact of RTN1 expression on amyloid peptide production

**Figure 4:**
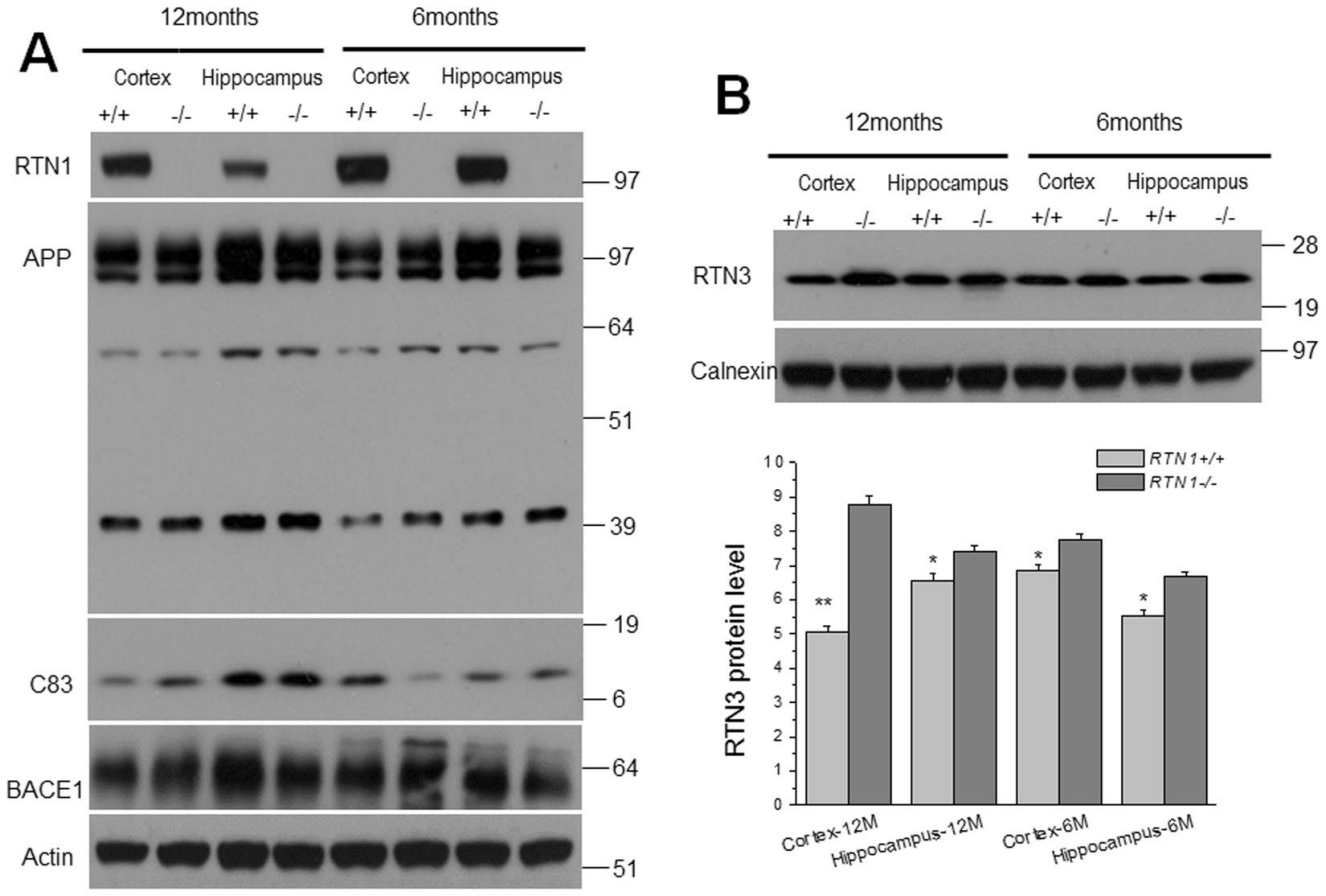
RTN1 deficiency increases RTN3 expression in brains. (**A**) Hippocampal protein lysates were prepared from 6- and 12-month-old WT and RTN1-null mice. Western blots were examined by antibody R418 to detect RTN1 expression and by A8717 to detect APP and its C-terminal fragments. No significant changes in APP-C83 levels were detected following RTN3 deletion. (**B**) A clear elevation of RTN3, as detected by R458, was observation on the western blot. Loading control was an antibody to calnexin. (**C**) Bar graphs showed relative levels of RTN3 over the loading control. (N = 4 independent experiments, *P < 0.05, **P < 0.01, Student’s *t*-test).

RTN proteins were found to interact with BACE1 and to negatively modulate its activity^24, 25^. To determine whether RTN1 deficiency affects BACE1 activity, we examined the expression of APP and its cleavage products in brain lysates. Notably, there were no clear changes in full-length APP or the α-secretase-cleaved APP fragment (APP-C83) upon RTN1 ablation (Fig. 4A). BACE1-cleaved APP fragments (APP-C99 and C89) were not readily separated and detected due to low levels of endogenous APP, and no obvious changes were observed upon significantly longer exposure (data not shown). The expression of BACE1 was also not obviously altered (Fig. 4B), which is unlike that seen in RTN3-null mice, where protein levels of BACE1 were markedly increased in six-month-old brains^12^. When we examined levels of other RTN members, we found that RTN3 protein levels were elevated in RTN1-null brains (Fig. 4B). This compensatory increase in RTN3 may offset the deleted RTN1 in the negative modulation of BACE1 levels and activity.

### Weak association between RTN1 and dystrophic neurites in AD

**Figure 5:**
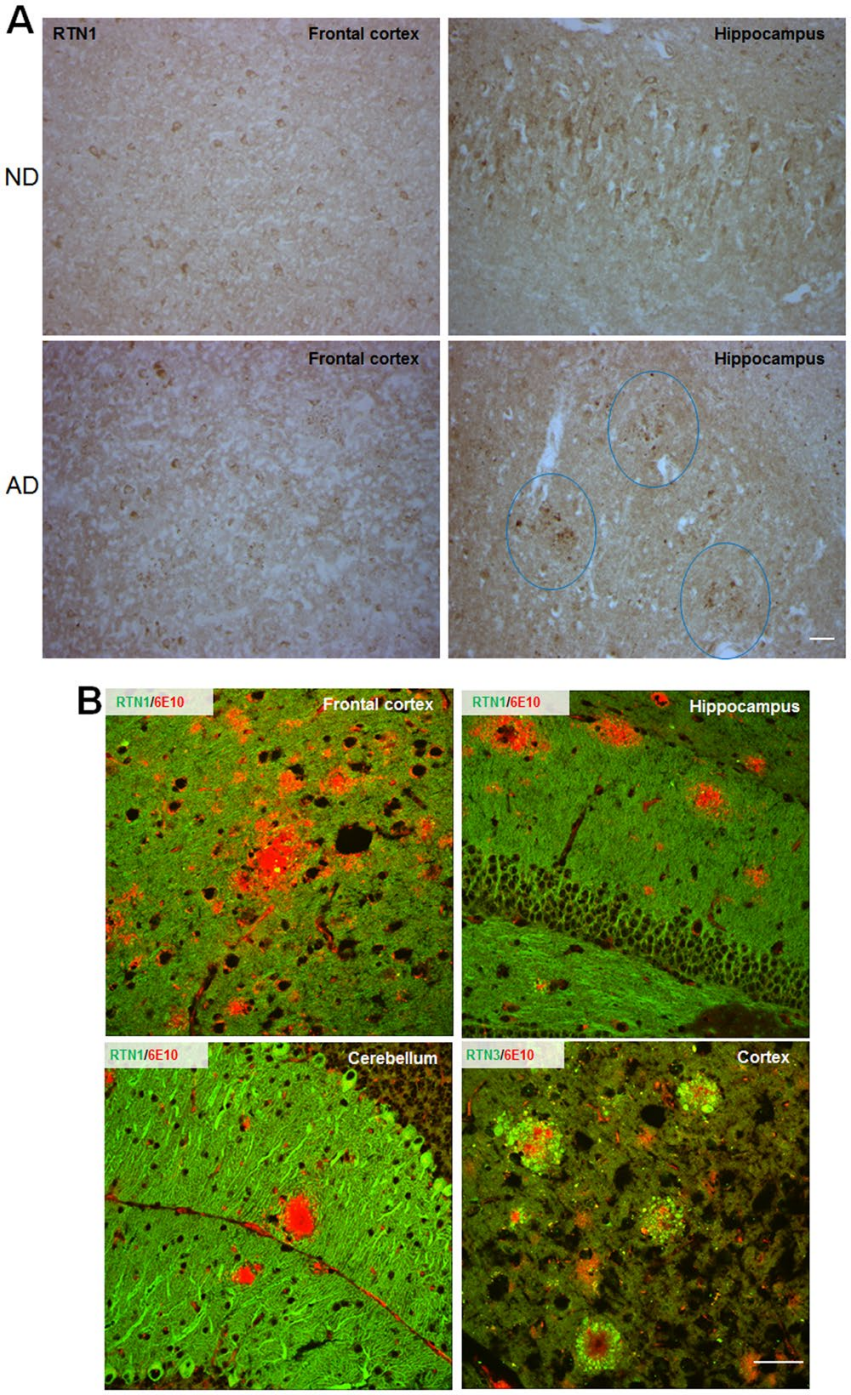
RTN1 and RTN3 differentially label dystrophic neurites in AD brains. (**A**) Brain sections from postmortem human (AD) or non-demented (ND) brains were labeled by antibody R418 in immunohistochemical staining. Strong neuronal expression of RTN1 was detected in ND samples. In AD brains, RTN1 appeared to be enriched in areas with amyloid plaques (circled). (**B**) Fixed brain samples from six-month-old Tg-APPswe/PSEN1ΔE9 mouse brains were detected by R418 for RTN1 (green), R458 for RTN3 (green), and 6E10 for amyloid plaques (red). Amyloid plaques from different brain regions, including the cerebellum, were selected. Unlike RTN3, RTN1 was weakly enriched in dystrophic neurites. Processes labeled by RTN1 were more evident than those labeled by RTN3. Scale bar is 30 µm.

Senile plaques in AD brains are recognized as core amyloid plaques surrounded by activated microglia, reactive astrocytes, and dystrophic neurites. RTN3 is found to be abundantly enriched in dystrophic neurites and overexpression of RTN3 is sufficient to develop similar dystrophic neurites^26, 27^. To determine whether RTN1 is also enriched in dystrophic neurites, we examined postmortem brain sections from AD patients and transgenic mice overexpressing Swedish mutant APP and PS1ΔE9 (Tg-APP/PS1ΔE9)^28^. We showed that RTN1 was normally expressed in neurons of non-demented control postmortem brains (Fig. 5A). However, in AD brains, it was clearly enriched near amyloid plaques (see circles in A, Fig. 5A), and mainly in the hippocampi, and it was not readily detected in frontal cortex in which amyloid plaques were also detected (data not shown). In fixed Tg-APP/PS1ΔE9 mouse brains, RTN1 was also found in dystrophic neurites surrounding amyloid plaques. Even though RTN1 is localized in dendrites of Purkinje cells and amyloid plaques were found in this region, RTN1 was weakly expressed in areas surrounding amyloid plaques (Fig. 5B). However, its family member RTN3 was significantly more enriched in dystrophic neurites (Fig. 5B), despite the fact that both proteins are similarly expressed by hippocampal and cortical neurons.

## Discussion

RTN1 was the first RTN member cloned as a neuroendocrine-specific protein^22^, initially characterized by Northern blot detection of RTN1 isoforms in neuroendocrine cell lines and a primary carcinoid lung tumor. Here we show that RTN1 is largely expressed by the nervous system, essentially by neurons in broad brain regions. Strikingly, RTN1 is richly distributed in dendrites of Purkinje cells, which was not reported previously. Such a distribution pattern is unique to RTN1 as RTN3, although expressed by Purkinje cells as well, does not label dendrites in particular. Since both RTN1 and RTN3 are expected to shape tubular ER^5^, such a distinct distribution of RTN1 and RTN3 in dendrites suggests that each RTN member is likely to exert a unique biological function in cells or neurons in addition to certain shared biochemical functions.

We demonstrate that RTN1 deficiency in mice does not cause obviously detectable defects in mouse fertility, growth, or behavior, which is similar to the deletion of its other family members such as RTN3 and RTN4. However, RTN1 and RTN3 do display differential effects on the pathological changes in AD brains. RTN1 deficiency has a lesser effect on BACE1 activity and Aβ levels, while RTN3 appears to have stronger modulation on BACE1 activity in neurons as RTN3 deficiency causes increased BACE1 protein levels^12, 24^. Consistently, BACE1-cleaved APP C-terminal fragment, APP-C99, is elevated in response to RTN3 deficiency but not RTN1 deficiency. In addition, increased amyloid deposition was observed in an AD mouse model with RTN3 deletion (Tg-APPswe/PS1ΔE9/RTN3^−/−^), even though RTN1 levels were slightly elevated upon RTN3 knockout^12^. Intriguingly, RTN1 deficiency also causes elevation of RTN3 protein levels (Fig. 3B), suggesting that expression of these two proteins is tightly regulated. Since the mouse *rtn1* gene is on chromosome 12C3, whereas the mouse *rtn3* gene is located at 14q23.1^3^, it is unclear why expression of these two proteins appears to be coupled.

More interestingly, RTN3 is abundantly enriched in dystrophic neurites that surround amyloid plaques found in the brains of AD patients and AD mouse models. On the contrary, RTN1 is only sparsely present in dystrophic neurites in the examined same samples. RTN4 is weakly expressed in neurons, and it was not readily in dystrophic neurites of human AD brains^26^. Such a differential effect cannot be explained by the shared function from their common RHD. One potential attribution is likely related to the unique sequence present in their N-terminal domains, and future studies will be needed to explore this area.

RTN1 does have unique biological functions, as demonstrated by others. During normal mouse development, RTN1-A appears to be the predominantly expressed RTN1 form, which is consistent with prior Northern blotting experiments^22^. Increased expression of RTN1 was found in tumors or carcinomas originating from neurodocrine cells^16, 29, 30^. In patients with diabetic nephropathy, the expression of RTN1-A is detected in the diseased kidneys from mice and humans, and this elevated expression inversely correlates with renal function^23^. RTN1-A is also found to interact with the intracellular calcium release channel ryanodine receptor 2 (RyR2) and increased expression of RTN1-A significantly reduced RyR2-mediated Ca^2+^ oscillations^31^. RTN1 proteins may undergo acetylation-deacetylation through activity of the zinc-dependent histone deacetylase (HDAC) enzyme HDAC8, as the C-terminal domain of RTN1 interacts with HDAC8^32^. Acetylation of RTN1 isoforms could induce ER stress-mediated apoptosis in neuroectodermal tumors^33^. Collectively, these reported functional roles appear to be unique to RTN1, and the available of RTN1-null mice will provide a useful tool to fully elucidate its biological functions *in vivo*.

In summary, we show that mice deficient in RTN1 are generally normal and that RTN1 is a predominantly expressed in the nervous system, mainly in neurons. In neurons, RTN1 is enriched in dendrites, and this appears to be unique to RTN1, but not RTN3, even though both proteins are likely to shape tubular ER via the RHD. This study also provides an opportunity to study why dendrites are more enriched in RTN1, but not RTN3. It is possible that this unique neuronal localization may differentiate the effects of RTNs in their contribution to AD pathogenesis, and future studies will be aimed at further mechanistic elucidation of specific RTN members in AD pathogenesis.
